# Parental contribution to trisomy in heterozygous androgenetic complete moles

**DOI:** 10.1038/s41598-020-74375-4

**Published:** 2020-10-13

**Authors:** Hirokazu Usui, Asuka Sato, Makio Shozu

**Affiliations:** 1 Department of Reproductive Medicine, Graduate School of Medicine, Chiba University, Chiba, 260-8670 Japan; 2 Department of Gynaecology, Chiba University Hospital, Chiba University, Chiba, 260-8670 Japan

**Keywords:** Cytogenetics, Development, Genetics, Molecular medicine, Pathogenesis, Risk factors, Cell biology, Cell division, Chromosomes

## Abstract

Complete hydatidiform moles (CHMs) comprise a proliferative trophoblastic disorder and are known to be androgenetic and diploid. Androgenetic CHMs are classified as having monospermic and dispermic origins. Rarely, some CHMs have other genetic constitutions, such as biparental diploid or tetraploid. Previous studies have shown the possibility that androgenetic heterozygous CHMs have an additional chromosome with high frequency. This study aimed to comprehensively analyse the molecular karyotyping of androgenetic dispermic CHMs and the parental contribution of their additional chromosomes. Single-nucleotide polymorphism arrays were performed with the genomic DNA of CHMs and patients. The B allele frequency and selected B allele frequency plotting of CHM were visualised. Among the 31 dispermic CHMs, eight showed trisomy and one showed double trisomy; of the 10 additional chromosomes, seven were of maternal original and three were of paternal origin. In addition, three disomic chromosomes comprised one maternal and one paternal chromosome, although these should theoretically have had two paternal chromosomes in the case of androgenetic CHMs. The subclassification of heterozygous CHMs, with or without maternal contribution, is a new approach and could be a candidate indicator of gestational trophoblastic neoplasia risk.

## Introduction

Hydatidiform moles are characterised by trophoblastic proliferation and swollen villous structures, and are mainly classified into two categories, complete hydatidiform moles (CHMs) and partial hydatidiform moles (PHMs). In almost all CHMs, the genome is androgenetic and diploid (i.e. only originating from sperm(s)), whereas the genome in most PHMs is diandric monogynic triploid^1,2^. In rare cases, CHMs have other genetic constitutions, such as biparental diploid^3^ or tetraploid^4^. The risk of developing gestational trophoblastic neoplasia (GTN) is higher with androgenetic CHMs (15–20%) than with PHMs (1–4%)^5–7^. Thus, the precise diagnosis of CHM is necessary^2^. Androgenetic CHMs can be further divided into homozygous and heterozygous cases, which are derived from a single sperm and two sperms, respectively^1,2^. Recently, heterozygous CHMs were reported to be associated with a higher risk than homozygous CHM^8,9^.

Pathologists have utilised immunohistochemistry for p57KIP2, the product of the imprinted gene cyclin dependent kinase inhibitor 1C, to detect CHM^2,10^. The p57KIP2 protein is expressed in cytotrophoblasts and stroma cells only from maternal chromosomes. Accordingly, androgenetic CHMs would show negative results for p57KIP2 immunostaining, whereas PHMs and hydropic abortions would show positive staining^11^. Paradoxically, cases with p57KIP2-positive androgenetic CHMs have been reported, including a study by our group^12–14^. These androgenetic CHMs were trisomy and had a retained maternal chromosome 11^12–14^. These cases were all heterozygous CHM but not homozygous CHM^12–14^.

Single-nucleotide polymorphism (SNP) array analysis was recently introduced to determine the genetic constitution of CHM, PHM, and non-molar villous tissues across whole chromosomes^14–17^. Using SNP array analysis, we reported that androgenetic heterozygous CHMs are of dispermic origin and that they do not originate from abnormal diploid sperm^17^. In addition, some of their chromosomes demonstrated trisomy^17^. The frequency of trisomy in androgenetic heterozygous CHMs has not been determined, although there have been a few case reports including p57KIP2-positive androgenetic CHM^12–14,18–20^. This study aimed to comprehensively analyse the genetic constitution of androgenetic dispermic CHMs and the parental origin of their additional chromosomes using SNP array data. Further, we tried to investigate the mechanism underlying androgenetic dispermic CHM development.

## Results

### Determination of genetic constitution of hydatidiform moles by STR analysis

Among 443 patients enrolled into the molecular diagnostic study of HMs, we identified 33 androgenetic dispermic CHMs, 236 androgenetic monospermic CHMs, 67 diandric monogynic triploid PHMs, two monoandric digynic triploid conceptuses, and 105 biparental diploid abortions, by STR polymorphism analysis. Two androgenetic heterozygous CHMs could not be used for SNP array analysis because of the scant amount of the sample.

### Hypothetical B allele frequency (BAF) and selected BAF

**Figure 1:**
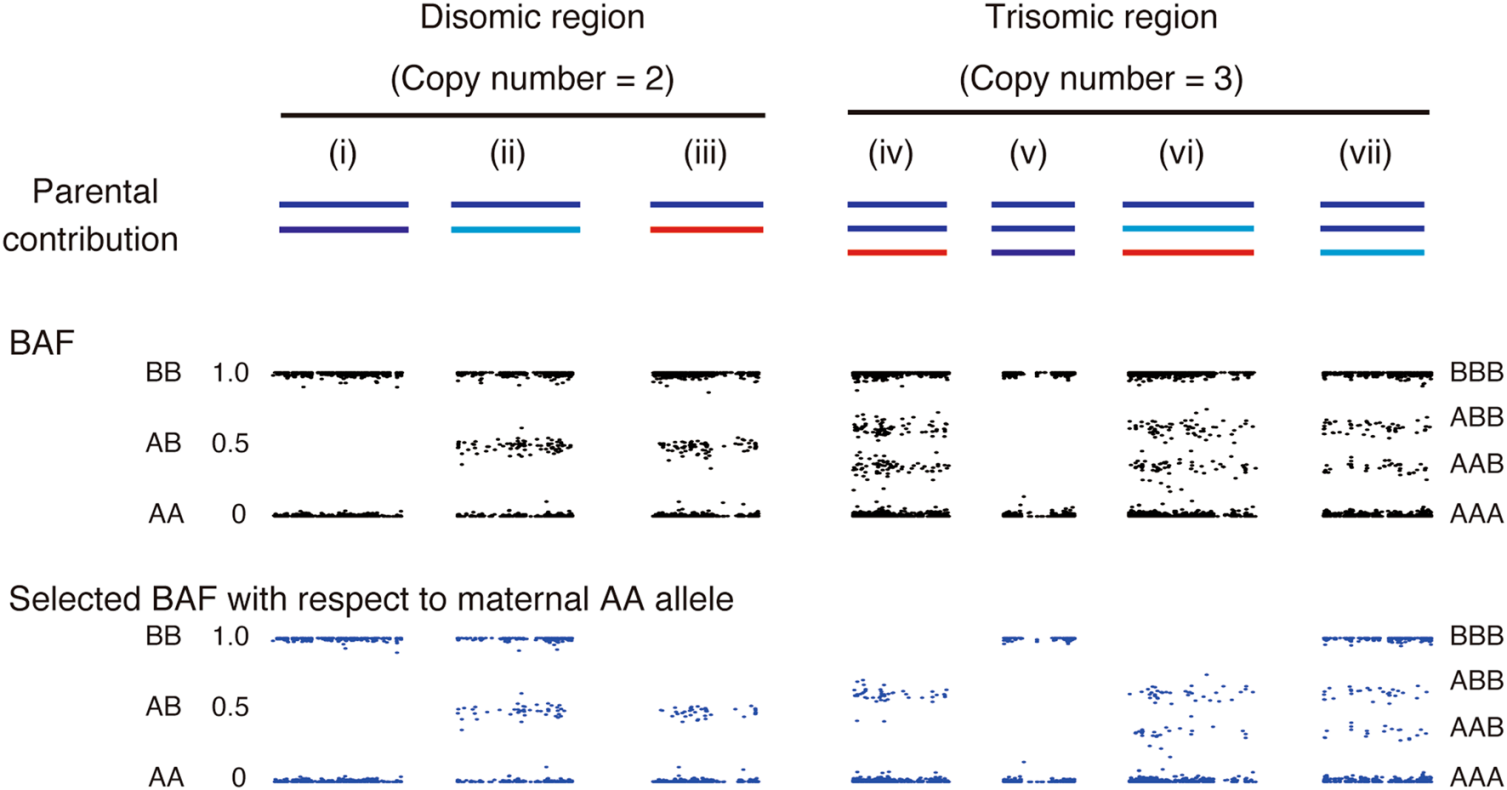
Relationship between chromosomal constitution, BAF, and selected BAF plotting. The presumed combinations of BAF and selected BAF plotting are presented. Blue and light blue regions are of paternal origin. Red regions are of maternal origin. *BAF* B allele frequency.

Hypothetical BAF plotting in regions with various chromosomal constitutions is depicted in Fig. 1. In disomic regions, BAF plotting showed two (0 and 1; AA and BB) or three lines (0, 0.5, 1.0; AA, AB, and BB) for regions in homozygous or heterozygous state, respectively. In trisomic regions, BAF plotting presented four lines (0, 0.33, 0.66, and 1.0 representing AAA, AAB, ABB, and BBB, respectively). When all three chromosomes were identical, two lines were present (0 and 1; AAA and BBB, respectively) as shown in Fig. 1(v). For diploid androgenetic CHMs, BAF values for loci in which the mother had the genotype AA, were selected (Table 1). We termed the BAF in these selected loci ‘selected BAF.’ When the regions included maternal contribution, the selected BAF plotting of the regions resulted in the loss of plots with BAF value 1.0 (Fig. 1(iii,iv,vi).

**Table 1:** Selected BAF plotting calling schema.

Illumina SNP ID	Mole BAF	Mole alleles	Maternal BAF	Maternal allele	Loci selected by maternal AA allele
exm870797	0.9841	BB	0.9865	BB	
exm2271913	0.4887	AB	0.4551	AB	
exm1163669	0.9737	BB	0.9901	BB	
exm151810	0.4712	AB	0.4921	AB	
exm363078	0.9788	BB	0.4917	AB	
exm1588790	0.9921	BB	0.4467	AB	
**exm717089**	**0.0089**	**AA**	**0.0002**	**AA**	**Selected**
**exm1436659**	**0.0058**	**AA**	**0.001**	**AA**	**Selected**
exm747290	0.4611	AB	0.9738	BB	
exm2265754	0.0042	AA	0.987	BB	
**exm779139**	**0.5202**	**AB**	**0.0056**	**AA**	**Selected**
exm1083876	0.0022	AA	0.4597	AB	
**exm-rs6929796**	**0.585**	**AB**	**0.0076**	**AA**	**Selected**
**exm1021627**	**0.9553**	**BB**	**0.0156**	**AA**	**Selected**
**exm44591**	**0.9822**	**BB**	**0.0233**	**AA**	**Selected**
exm2259465	0.0043	AA	0.4864	AB	
exm2267990	0.0102	AA	0.9968	BB	
exm-rs10472828	0.467	AB	0.9919	BB	

This table was extracted from the InfiniumExome-24v1 array output for both the mole and patient of case HM01. Bold lanes indicate the loci with respect to maternal AA alleles. The selected BAF plotting (by maternal AA) is depicted by the BAF of the loci with respect to maternal AA alleles.

*BAF* B allele frequency.

### SNP array analysis

**Figure 2:**
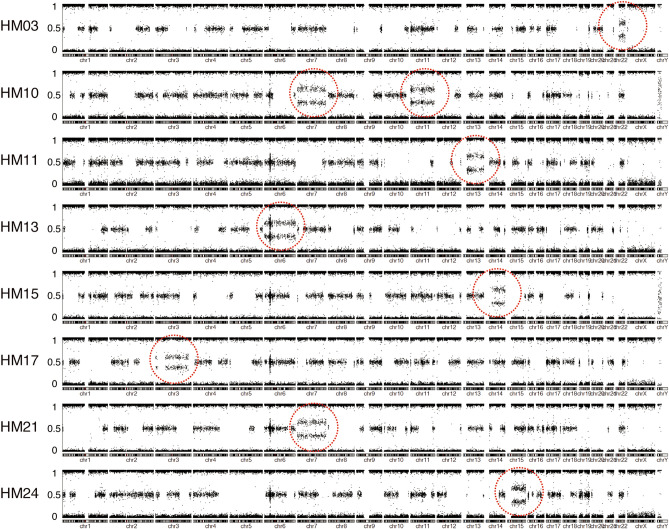
BAF plotting of the sample including autosomal trisomic chromosomes. All samples showed segmental homozygosity throughout the autosomal chromosomes. In dotted circles, nine chromosomes represent the trisomic character, indicating the four lines AAA, AAB, ABB, and BBB. Trisomic chromosomes are presented in Table 2. BAF: B allele frequency.

Twelve CHM samples had previously been analysed using the CytoSNP-12 array as described in the previous report (HM01–12)^17^. Patients and their corresponding CHMs (22 samples) were analysed using the InfiniumExome-24v1 array (HM03, HM10, HM11, and HM13–31). Call rates and GenCall scores are shown in the Supplementary Table S1 online. BAF and log R ratio (LRR), plotted using the InfiniumExome-24 array data, are shown in Supplementary Fig. S1 online. All BAF plots demonstrated segmental homozygosity across whole genomes, reflecting the meiotic recombination during spermatogenesis. All LRRs in autosomal chromosomes were close to 0, which indicated a copy number of two and diploidy. The BAF plotting of CHMs with trisomy of autosomal chromosomes is shown in Fig. 2. The plotting of trisomic chromosomes presented four lines (Fig. 2).

The mitochondrial genotypes of SNP loci between patients and their moles were all identical (Supplementary Table S2 online). Among 208 mitochondrial loci of the InfiniumExome-24v1 (Illumina, Inc.) array, 31 loci were informative, which showed the different genotypes among the samples. Two pairs of cases (HM22/HM29 and HM10/HM21) were identical for all mitochondrial loci based on the results of the SNP array (Supplementary Table S2 online).

### Estimated karyotypes

The information for BAF and LRR plots of whole chromosomes including the sex chromosomes (Supplementary Fig. S1 online) was used to estimate the molecular karyotypes, as shown in Table 2. In addition, we confirmed the number of each chromosome using the histograms of the LRR distribution. The medians and means of trisomic chromosome LRRs shifted to the right (larger) compared to those of disomic chromosomes (Supplementary Fig. S2 online and Supplementary Table S3 online).

**Table 2:** List of androgenetic heterozygous complete hydatidiform moles.

ID	Array type	Estimated Karyotype^#^	Origin of additional chromosome(s)	Deletion of paternal chromosome	Post-molar outcome
HM01	C	46,XX		*	SR
HM02	C	46,XX		*	SR
HM03	C, E	47,XY, + 22	22, mat		SR
HM04	C	46,XY		*	GTN
HM05	C	47,XYY	Y, pat	*	GTN
HM06	C	46,XY		*	GTN
HM07	C	46,XX		*	SR
HM08	C	46,XY		*	SR
HM09	C	46,XX		*	SR
HM10	C, E	48,XX, + 7, + 11	7, mat.; 11, mat	Chr. 4, mat/pat	SR
HM11	C, E	47,XY, + 13	13, mat	Chr. 6, mat/pat	SR
HM12	C	46,XX			SR
HM13	E	47,XY, + 6	6, mat		SR
HM14	E	46,XX			SR
HM15	E	47,XX, + 14	14, pat		GTN
HM16	E	46,XY			GTN
HM17	E	47,XY, + 3	3, pat		SR
HM18	E	46,XY			SR
HM19	E	46,XX			GTN
HM20	E	46,XX			SR
HM21	E	47,XY, + 7	7, mat		SR
HM22	E	46,XX			GTN
HM23	E	46,XY			SR
HM24	E	47,XY, + 15	15, mat	Chr. 2, mat/pat	SR
HM25	E	46,XY			SR
HM26	E	46,XY			SR
HM27	E	46,XY			SR
HM28	E	46,XY			SR
HM29	E	46,XY			SR
HM30	E	46,XY			SR
HM31	E	46,XY			SR

*SR* spontaneous remission; *GTN* gestational trophoblastic neoplasia; *Chr.* chromosome; *mat.* maternal; *pat.* paternal; *mat/pat.* one maternal chromosome and one paternal chromosome.

Array type: C, Illumina Human CytoSNP-12; E, InfiniumExome-24v1.

*This was not determined because we could not perform the maternal SNP array analysis.

^#^Actual karyotypes of trisomic cases could be almost accurately determined for comparison of the BAF and LRR of disomic and trisomic chromosomes. Conversely, the possibility of tetraploidy could not be excluded strictly in the cases with euploid heterozygous CHM.

Twenty-two cases were euploid. Among them, the karyotypes of 9 and 13 cases were 46,XX and 46,XY, respectively. Nine cases were aneuploid. One case had a karyotype of 47,XYY, which would have originated from 24,XY and 23,Y sperms. The remaining eight CHMs were aneuploid in autosomal chromosomes, showing additional chromosomes (Fig. 2 and Table 2). HM10 showed double trisomy of chromosome 7 and 11 (Fig. 2).

### Parental contribution of additional chromosomes

**Figure 3:**
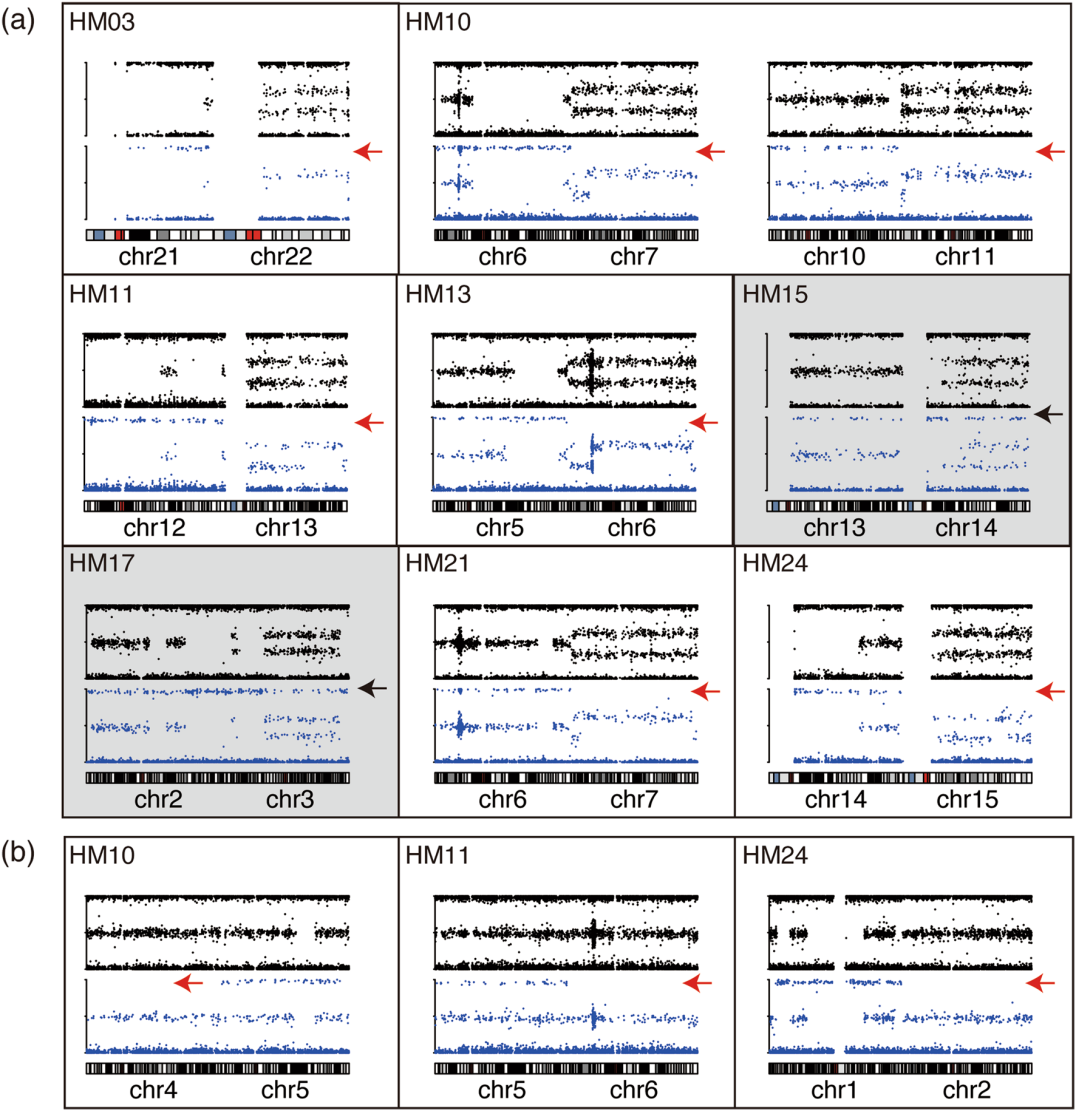
BAF and selected BAF plotting of chromosomes with abnormal constitution among trisomic chromosomes (**a**) and disomic chromosomes with maternal contribution (**b**). Upper and lower charts are BAF and the selected BAF, respectively. Red and black arrows show the loss of and remaining BAF near 1.0, respectively. A grey background indicates that additional chromosomes are of paternal origin. *BAF* B allele frequency.

We performed ‘selected BAF’ plotting analysis to unveil the parental origins of additional chromosomes. The BAF and selected BAF plots of nine additional chromosomes are shown in Fig. 3a. Additional chromosomes were of maternal origin for seven chromosomes (HM03, chr. 22; HM10, chr.7 and 11; HM11, chr. 13; HM13, chr. 6; HM21, chr. 7; HM24, chr. 15) because the selected BAF plots near 1.0 were lost as same pattern as Fig. 1(iv) or (vi). In contrast, the selected BAF plots near 1.0 for additional chromosome 14 of HM15 and chromosome 3 of HM17 remained, which indicated that the additional chromosomes were of paternal origin (Fig. 1(vii)). Mitochondrial SNPs were identical between the patients and the CHM in HM15 and HM17 (Supplementary Fig. S2 online). Thus, we concluded that the trisomy of HM15 and HM17 were of paternal origin.

### Biparental disomic chromosomes in androgenetic heterozygous CHM

The selected BAF plotting for disomic chromosomes should not be changed in terms of the signature for cases with both chromosomes of paternal origin (Fig. 1(i,ii)). However, three chromosomes (HM10, chr.4; HM11, chr. 6; HM24, chr. 2) showed the loss of plots near 1.0, indicating that the disomic chromosomes would be biparental (Fig. 3b and Table 2).

### Relationship between the incidence of GTN and additional maternal chromosomes

In this series of cases, the incidence of GTN was 23% (7/31). Stratified by the existence of maternal contribution, the incidences were 0% (0/6) and 28% (7/25) in the presence and absence of maternal contribution, respectively.

### Frequency of aneuploidy in androgenetic homozygous CHMs

If a maternal or a second paternal chromosome was retained in a monospermic diploid CHM, the HMs could erroneously be classified as heterozygous diploid androgenetic CHMs. We reviewed the STR electropherograms of 31 heterozygous and 236 homozygous androgenetic CHMs. Among 16 loci of PowerPlex 16 system, the medians and ranges of two allelic loci of patients’ blood and CHMs in androgenetic heterozygous CHMs were 12 (8–14) and 7 (3–11), respectively (Supplementary Table S4 online). The minimum number of two allelic loci in heterozygous CHM was 3. There were no homozygous CHM with two loci that had two alleles. Only one case of homozygous CHM had one two-allelic locus, which was proven as monospermic CHM with tetrasomy of sex chromosomes, 48,XXYY^17^. Thus, the frequency of aneuploidy of androgenetic homozygous CHMs was one per 236 in our series. The frequencies of trisomy were significantly different (monospermic CHM; 0.4% [1/236] vs. dispermic CHM; 29% [9/31], *P* = 0.01, Fisher’s exact test).

### Ploidy analysis of androgenetic homozygous CHMs

Correctly identifying aneuploidy in a homozygous diploid CHM may be difficult using STR analysis only, because some loci may show a change in only peak-heights. To determine the existence of aneuploid chromosomes in androgenetic monospermic CHM, we next analysed the Gene Expression Omnibus data (GSE54948), which were from the androgenetic homozygous CHM genomes of Japanese patients^21^. We analysed the BAF and LRR plotting on each chromosome among 97 CHM cases. All chromosomes except one chromosome X (CHM035) maintained LRR values near 1.0, indicating that aneuploid monospermic CHM would be rare (Supplementary Fig. S3 online and Supplementary Table S5 online). The karyotype of aneuploid CHM was estimated to be 48,XXXX (Supplementary Fig. S3 online).

## Discussion

This study demonstrated that androgenetic dispermic CHMs show a high rate of aneuploidy, with a frequency of 29% (9/31). To the best of our knowledge, this is the first comprehensive study using a large number of samples to clarify the molecular karyotyping of androgenetic dispermic CHMs and their parental origin. In our previous study to determine the origin of heterozygous androgenetic CHM, three cases among 12 dispermic CHMs had chromosomes with trisomy^17^. In this study, selected BAF plotting could discriminate additional chromosome origins, namely maternal or paternal. Both types of extra chromosomes were observed. Recently, we reported two cases showing positive p57KIP2 immunostaining (HM10 and HM16 in this study), one of which (HM10) retained maternal chromosome 11^14^. In addition, two other case reports demonstrated the retention of maternal chromosomes in androgenetic CHMs, in which the cases showed positive p57KIP2 immunostaining and chromosome 11 trisomy^12,13^. All of the androgenetic CHMs with retained maternal chromosomes were heterozygous and dispermic^12–14^. Other than trisomy 11, two cases of androgenetic heterozygous CHM with trisomy 9 were reported^18,19^. This study and the reported cases imply that aneuploidy in dispermic CHMs is not rare.

The aneuploid monospermic CHM was rare. We detected only one case of monospermic aneuploid CHM among our samples. The molecular karyotype of the monospermic aneuploid CHM as determined by SNP analysis was 48,XXYY, which was derived from fertilisation by a single 24,XY sperm^17^. Dube et al. reported a case of androgenetic homozygous (monospermic) CHM with additional chromosome 13 based on FISH and STR analyses^22^. We analysed the largest dataset of SNP array of androgenetic homozygous CHM. Among 97 monospermic CHMs, only one chromosome showed aneuploidy as tetrasomy of chromosome X. The data of median LRR per chromosomes indicated little chance of trisomy or tetrasomy in monospermic CHM, although the strict determination of ploidy such as diploidy, triploidy, and tetraploidy, cannot be possible without classic karyotyping or FISH.

**Figure 4:**
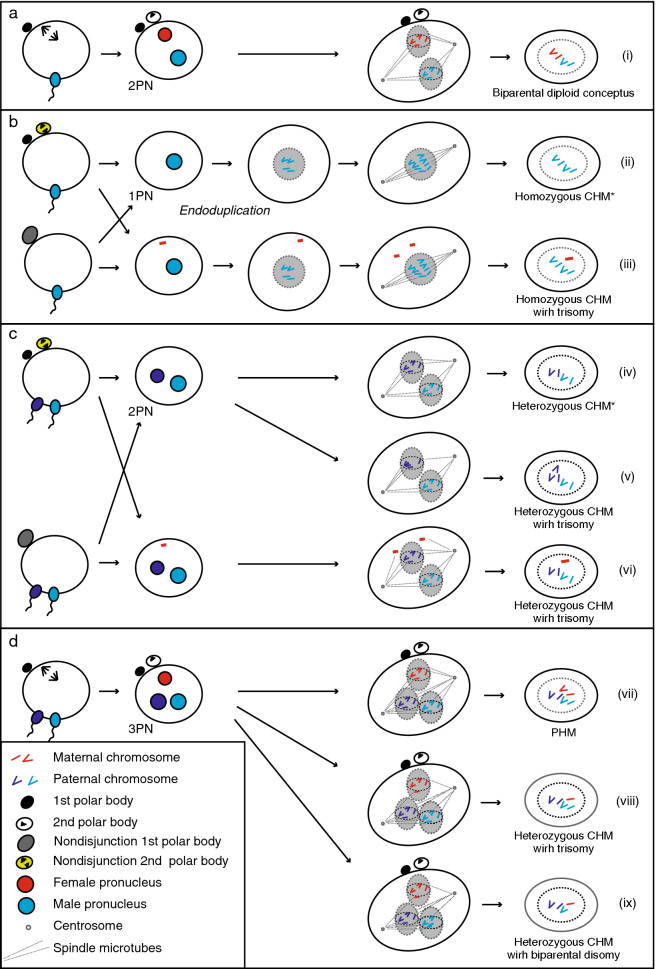
Possible developmental scenarios of dispermic fertilisation to hydatidiform moles. (**a**) Typical one sperm fertilisation results in growth to 2PN and normal diploid cells. (**b**) Typical one sperm fertilisation and second meiotic failure of the oocyte results in 1PN. Endoduplication would result in androgenetic homozygous (monospermic) CHMs. (**c**) Dispermic fertilisation and the second meiotic failure of the oocyte result in 2PN. The first equal division would produce androgenetic heterozygous (dispermic) CHMs (iv). (**d**) Dispermic fertilisation results in growth to 3PN. Division with two centrioles would result in diandric monogyny partial hydatidiform moles (vii). Uneven division would result in androgenetic heterozygous (dispermic) CHMs with additional maternal chromosomes (viii). *CHM* complete hydatidiform mole; *PHM* partial hydatidiform mole; *PN* pronuclei.

The key steps in the development of HMs are the penetration of sperm(s) into an empty oocyte and the first division after fertilisation (Fig. 4). In normal fertilisation, one sperm penetrates an oocyte, followed by completion of the second meiosis of the oocyte. After in vitro fertilisation, two pronuclei (2PN) can be observed at day 1^23^. Later, the zygote divides into two identical cells (two-cell stage) (Fig. 4a). The mechanism of androgenetic CHM development, and maternal nucleus loss, has been known for a long time. Recently, *MEI1*, *TOP6BL/C11orf80*, and *REC114* were reported as candidate causative genes by screening patients with recurrent androgenetic CHMs^24^. In *Mei1*-deficient female mice, 8% of the oocytes lose all their chromosomes by extruding them with the spindles into the first polar body. However, *Mei1*^−/−^ oocytes are capable of fertilisation, and 5% produce androgenetic zygotes^24^. These types of developmental mechanisms are summarised as the failure of the first or second meiosis in oocytes, resulting in monospermic and dispermic androgenetic CHMs (Figs. 4b(ii),ci(v)). Additional paternal chromosomes in this study (case #5, #15, and #17) might be explained by the disomic sperm contribution or unequal separation after dispermic fertilisation (Fig. 4c(v)).However, this mechanism could not explain the retained maternal chromosomes in heterozygous androgenetic CHMs.

The origins of maternal chromosomes might be postulated as incomplete extrusion of the diploid polar body of oocytes (Fig. 4c(vi)) or three pronuclei (3PN) (Fig. 4d)^25^. After fertilisation with two sperms, the separation of all chromosomes from the three nuclei by two centrioles would result in diandric monogyny triploid PHM (Fig. 4d(vii)). Biparental disomy (Fig. 4d(vii)) and trisomy with retained maternal chromosome (Fig. 4d(ix)) might be explained by the 3PN model.

The relationship between the developing potential of GTN and genetic constitutions has been discussed for several decades. The risk of developing GTN is higher for dispermic CHMs than for monospermic CHM^8,9^. Our previous study did not support this idea (monospermic CHM; 14%[29/204] vs. dispermic CHM; 21%[6/28], *P* = 0.40, Fisher’s exact test)^6^. To date, the known risk factors for GTN are maternal age and pre-evacuated human chorionic gonadotropin levels^6^. No specific causative gene has been reported. In this study, GTN development was not found in any case with additional maternal chromosomes. The subclassification of heterozygous CHMs, with or without maternal contribution, is a new concept and could be candidate marker of GTN risk. The relationship between trisomy with maternal contribution and the incidence of GTN is preliminary and requires the further accumulation of data.

SNP arrays demonstrate an advantage to analyse molar and non-molar villous tissues. The discrimination between disomic and trisomic chromosomes is visually easy as three and four lines, respectively, are obtained. In cases with STR analysis, the peak heights of two allele loci on disomic chromosomes are almost even but show scattering in some cases^26^. SNP array can overcome this limitation via the utilisation of many SNP loci throughout the entire genome. Further, the combination of both patient and their villous samples, selected BAF, could determine the parental origins, similar to the principal of the SNP duo tool reported by Roberson et al^27^. The selected BAF analysis can thus add further information regarding molecular karyotyping.

The limitation of this study was the small sample size, although the number of analysed heterozygous CHMs was the largest reported to date. Further accumulation of data might uncover the frequency of trisomy in dispermic CHM and the developmental mechanism of heterozygous CHM. In addition, the personalised risk of GTN could be unveiled.

In conclusion, this study demonstrated that androgenetic dispermic CHMs might have a high frequency of trisomy, with both maternal and paternal origins. We also estimated the developmental mechanism of dispermic androgenetic CHMs with additional chromosomes, Additional maternal chromosomes might be associated with the developmental mechanism of androgenetic dispermic CHM. Future studies could provide further details regarding this topic.

## Methods

### Ethics approval and consent to participate

The studies were approved by the Biomedical Research Ethics Committee of the Graduate School of Medicine, Chiba University (Approval reference No. 789 and 884). Written informed consent was obtained from all patients before participation, in accordance with the Declaration of Helsinki.

### Samples and data collection

Between 2007 and 2018, 443 patients were enrolled in a molecular diagnosis study at the Graduate School of Medicine, Chiba University, because they were suspected with molar pregnancy mainly based on sonographic findings^6^. Villous tissues and blood samples were collected from the patients. Pathological evaluation of products of conception was performed in the pathological department of Chiba University Hospital.

Finally, 31 patients with androgenetic dispermic CHMs diagnosed by STR analysis (described later) were enrolled in this study. Clinical data regarding GTN development were collected from the medical charts of the patients.

### DNA preparation and STR polymorphism analysis

The genomic DNA of villous tissue and blood was extracted using the QIAamp DNA Mini Kit (Qiagen, Hilden, Germany) in accordance to the manufacturer’s instructions^17^. STR polymorphism analysis was performed using the PowerPlex 16 or PowerPlex 16 HS System (Promega, Madison, WI), as described previously^17^. If one or more loci of the villous tissue did not share the allele(s) with any maternal alleles, they were classified as androgenetic. If the androgenic CHM had at least one locus with two different alleles, it was considered androgenetic dispermic CHM. Androgenic CHMs with only one allele at all loci were classified as androgenetic monospermic CHM.

### SNP array analysis and B allele frequencies

Genomic DNA of the 31 androgenetic dispermic CHMs was analysed with the Illumina Human CytoSNP-12 v2.1 BeadChip array or InfiniumExome-24v1, in accordance to the protocol provided by the manufacturer (Illumina, Inc., San Diego, CA)^17^. Raw data were normalised in GenomeStudio (Illumina, Inc.) using the information contained within the array. BAF and LRR were calculated using GenomeStudio. For androgenetic CHMs, BAF values for loci where the genotype in the maternal sample was AA were selected using the filter function in GenomeStudio as ‘.GType = AA’ for the corresponding patients (maternal; Table 1). We termed the BAF plotting with these selected loci ‘selected BAF plotting’.

### Visualisation of BAF and selected BAF plotting

Visualisation of BAF, selected BAF, and LRR was performed using the karyoploteR package version 1.10.4 (https://bioconductor.org/packages/release/bioc/vignettes/karyoploteR/inst/doc/karyoploteR.html) in R software (https://www.R-project.org).

### Mitochondrial SNP identity

Evaluations of mitochondrial SNP identity between patients and their moles were performed using InfiniumExome-24v1 (Illumina, Inc.) data, which had 208 mitochondrial SNP loci. The data of mitochondrial ‘GType’ on GenomeStudio (Illumina, Inc.) were exported. Then, the loci showing the identical genotype among all samples were excluded. The samples were ordered and sorted in accordance with the genotypes of each locus using Excel software (Microsoft, Corp., WA, USA).

### Analysis of Gene Expression Omnibus data

The Gene Expression Omnibus data (GSE54948) were retrieved from the National Centre for Biotechnology Information website (https://www.ncbi.nlm.nih.gov/geo/query/acc.cgi?acc=GSE54948). The array platform used was Illumina Human1M-Duov3 DNA Analysis BeadChip (GPL18247). The processed data and the platform information were analysed using R software.

## Supplementary information

Supplementary Figures.

Supplementary Table S1.

Supplementary Table S2.

Supplementary Table S3.

Supplementary Table S4.

Supplementary Table S5.

## Data Availability

The datasets generated and analysed during the current study are available from the corresponding author upon reasonable request.

## References

[1] Seckl MJ, Sebire NJ, Berkowitz RS (2010). Gestational trophoblastic disease. Lancet.

[2] Hui P, Buza N, Murphy KM, Ronnett BM (2017). Hydatidiform moles: genetic basis and precision diagnosis. Annu. Rev. Pathol..

[3] Murdoch S (2006). Mutations in NALP7 cause recurrent hydatidiform moles and reproductive wastage in humans. Nat. Genet..

[4] Sundvall L (2013). Tetraploidy in hydatidiform moles. Hum. Reprod..

[5] Savage PM (2013). The relationship of maternal age to molar pregnancy incidence, risks for chemotherapy and subsequent pregnancy outcome. J. Obstet. Gynaecol..

[6] Usui H (2018). Gestational trophoblastic neoplasia from genetically confirmed hydatidiform moles: prospective observational cohort study. Int. J. Gynecol. Cancer.

[7] Albright BB (2020). Gestational trophoblastic neoplasia after human chorionic gonadotropin normalization following molar pregnancy: a systematic review and meta-analysis. Obstet. Gynecol..

[8] Khawajkie Y (2020). Comprehensive analysis of 204 sporadic hydatidiform moles: revisiting risk factors and their correlations with the molar genotypes. Mod. Pathol..

[9] Zheng XZ (2020). Heterozygous/dispermic complete mole confers a significantly higher risk for post-molar gestational trophoblastic disease. Mod. Pathol..

[10] Fukunaga M (2002). Immunohistochemical characterization of p57(KIP2) expression in early hydatidiform moles. Hum. Pathol..

[11] Banet N (2014). Characteristics of hydatidiform moles: analysis of a prospective series with p57 immunohistochemistry and molecular genotyping. Mod. Pathol..

[12] Fisher RA (2004). Complete hydatidiform mole retaining a chromosome 11 of maternal origin: molecular genetic analysis of a case. Mod. Pathol..

[13] McConnell TG, Norris-Kirby A, Hagenkord JM, Ronnett BM, Murphy KM (2009). Complete hydatidiform mole with retained maternal chromosomes 6 and 11. Am. J. Surg. Pathol..

[14] Usui H, Sato A, Ota M, Ikeda JI, Shozu M (2020). Androgenetic complete hydatidiform moles with p57KIP2-positive immunostaining. Am. J. Clin. Pathol..

[15] Xie Y (2016). Single nucleotide polymorphism-based microarray analysis for the diagnosis of hydatidiform moles. Mol. Med. Rep..

[16] Carson JC (2018). Diploid/triploid mixoploidy: a consequence of asymmetric zygotic segregation of parental genomes. Am. J. Med. Genet. A.

[17] Usui H, Nakabayashi K, Maehara K, Hata K, Shozu M (2019). Genome-wide single nucleotide polymorphism array analysis unveils the origin of heterozygous androgenetic complete moles. Sci. Rep..

[18] Suzuki M, Itakura A, Ino K, Yamamoto T (2007). A triplet pregnancy featuring a 47, XY+9 heterozygous complete mole coexisting with two fetuses at 9 weeks. Acta Obstet. Gynecol. Scand..

[19] Kan ASY (2018). A fetus coexisting with a complete hydatidiform mole with trisomy 9 of maternal origin. J. Obstet. Gynaecol. Res..

[20] Ronnett BM (2018). Hydatidiform moles: ancillary techniques to refine diagnosis. Arch. Pathol. Lab. Med..

[21] Tahira T (2014). A definitive haplotype map of structural variations determined by microarray analysis of duplicated haploid genomes. Genom. Data.

[22] Dube V (2010). Androgenetic complete mole with trisomy 13: report of a case with microsatellite genotyping and review of the literature. Pathol. Res. Pract..

[23] Golubovsky M (2002). Paternal familial twinning: hypothesis and genetic/medical implications. Twin Res..

[24] Nguyen NMP (2018). Causative mutations and mechanism of androgenetic hydatidiform moles. Am. J. Hum. Genet..

[25] Golubovsky MD (2003). Postzygotic diploidization of triploids as a source of unusual cases of mosaicism, chimerism and twinning. Hum. Reprod..

[26] Kaku H, Usui H, Qu J, Shozu M (2016). Mature cystic teratomas arise from meiotic oocytes, but not from pre-meiotic oogonia. Genes Chromosomes Cancer.

[27] Roberson ED, Pevsner J (2009). Visualization of shared genomic regions and meiotic recombination in high-density SNP data. PLoS ONE.

