# Supplementary material for: Parental contribution to trisomy in heterozygous androgenetic complete moles
Source: Sci Rep. 2020 Oct 13;10:17137. doi: 10.1038/s41598-020-74375-4 (PMC7555529; doi:10.1038/s41598-020-74375-4)
Supplement: Supplementary file 1 — Supplementary Figures. [file 41598_2020_74375_MOESM1_ESM.pdf]

Supplementary information

Parental contribution to trisomy in heterozygous androgenetic complete moles

Hirokazu Usui[1,2], Asuka Sato[1,2], Makio Shozu[1,2]

[1] Department of Reproductive Medicine, Graduate School of Medicine, Chiba University

[2] Department of Gynecology, Chiba University Hospital, Chiba University

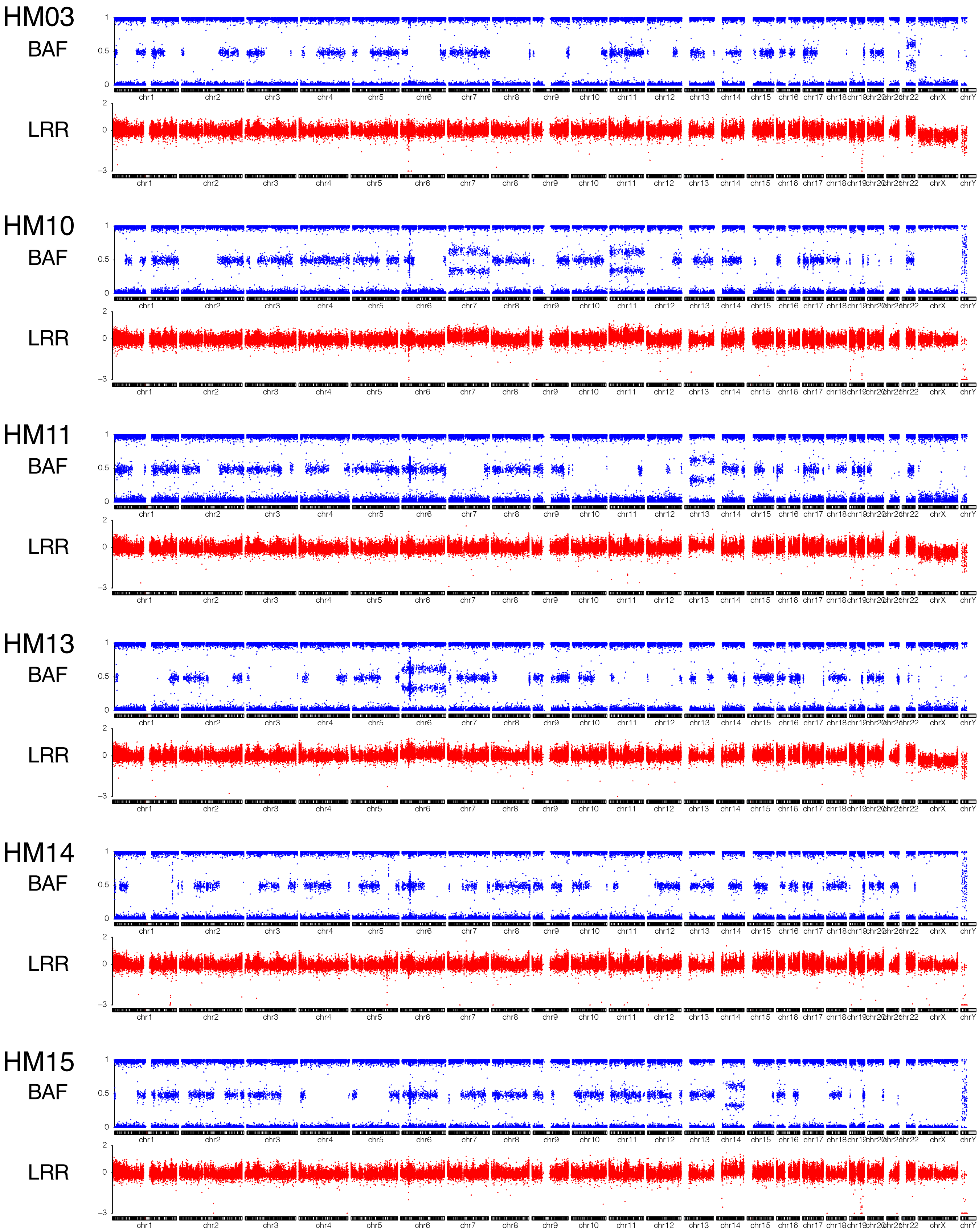

Supplementary Information Figure S1 (A)

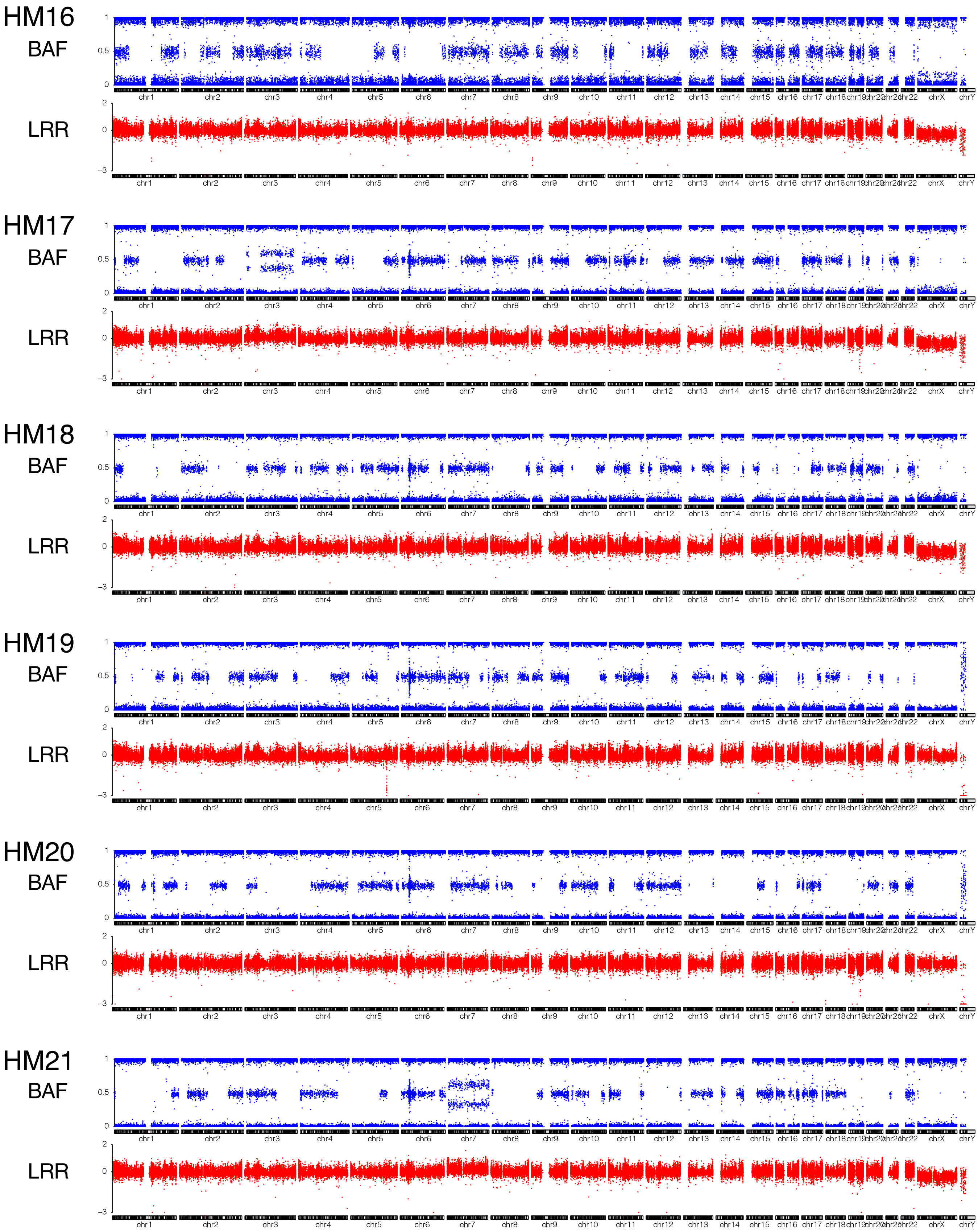

Supplementary Information Figure S1 (B)

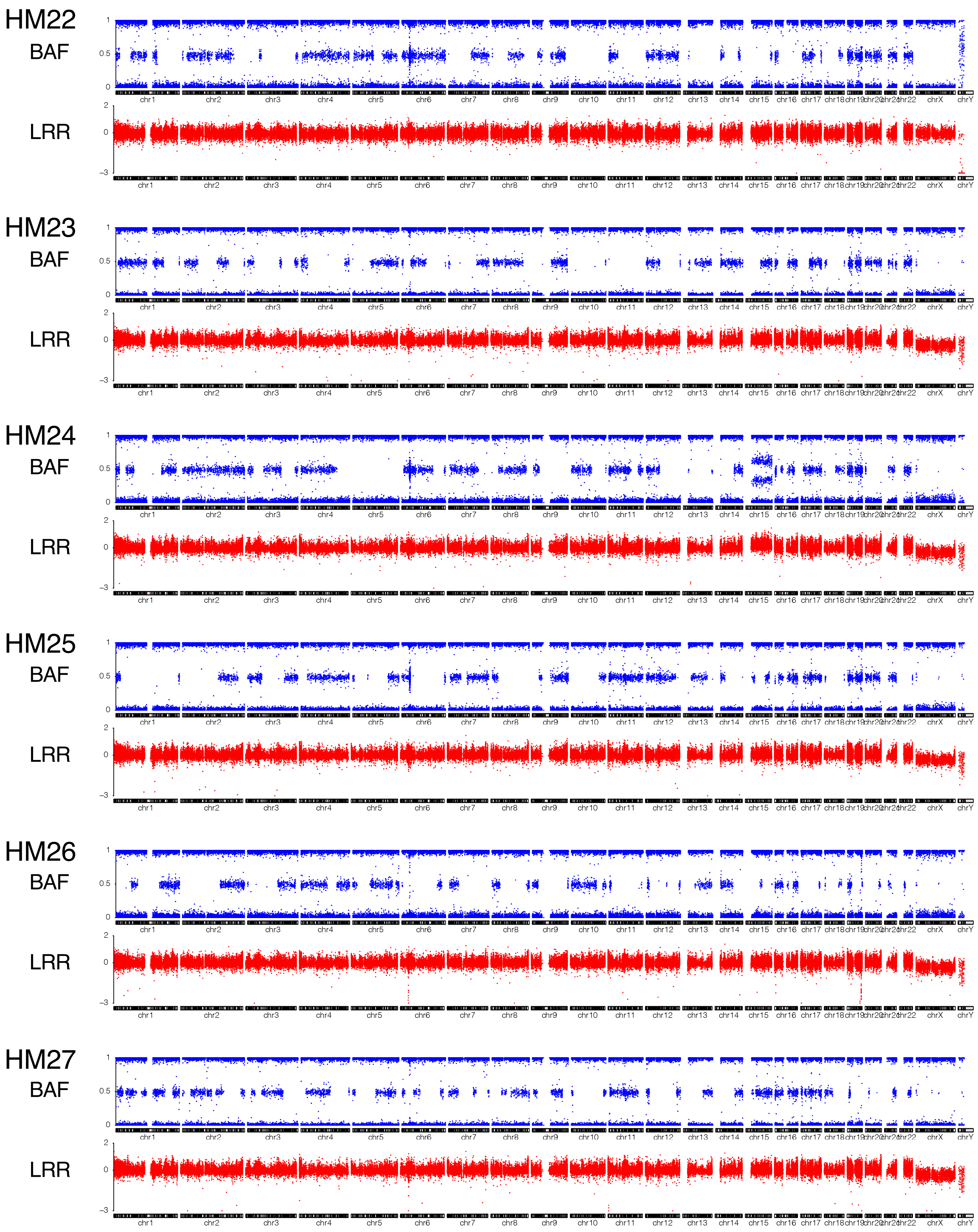

Supplementary Information Figure S1 (C)

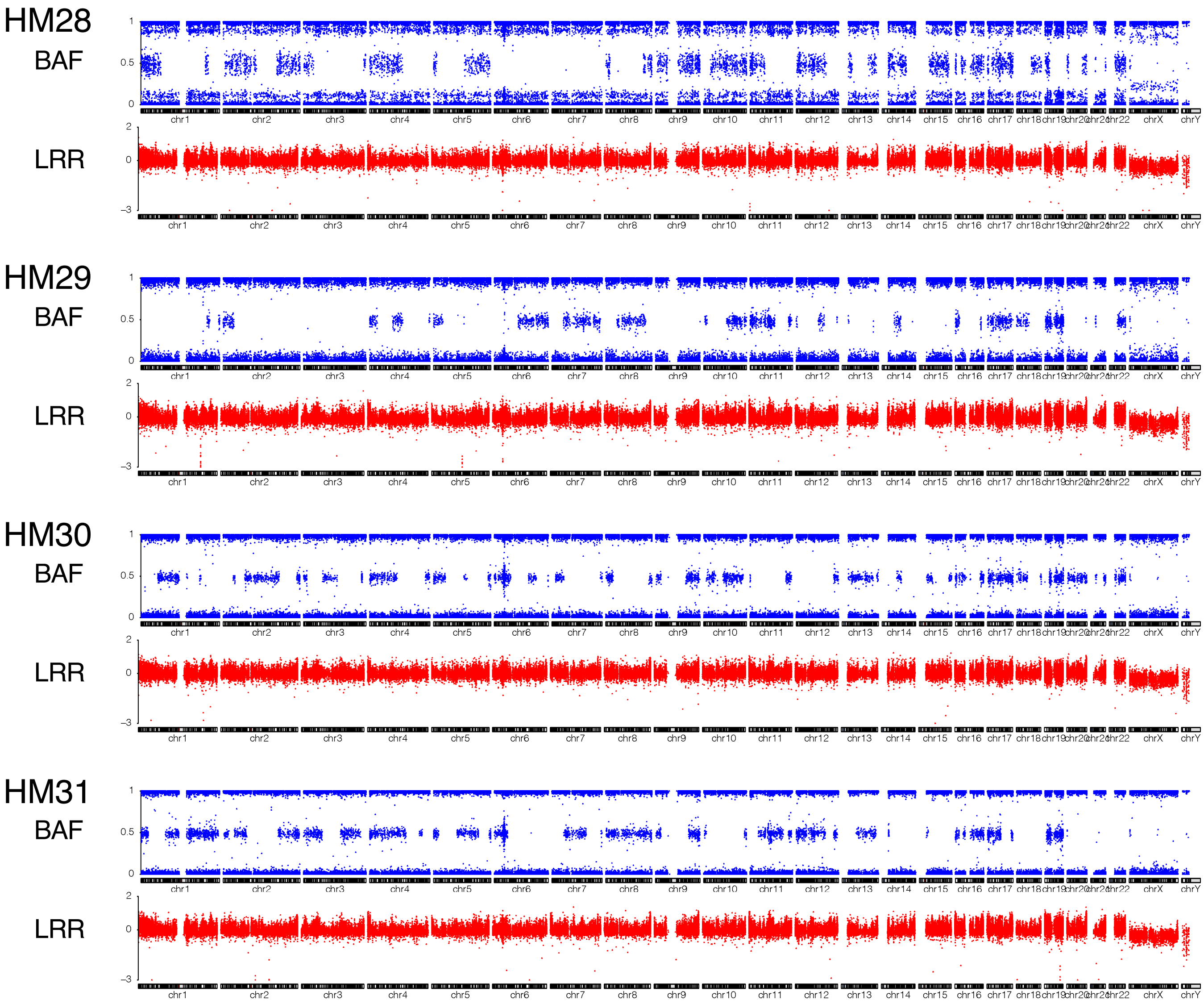

Supplementary Information Figure S1 (D)

Supplementary Figure S1

B allele frequency and log R ratio plotting using InfiniumExome-24 array data
(A) B allele frequency (BAF) and log R ratio (LRR) plotting of HM03, HM10, HM11, and HM13–15 cases. (B) BAF and LRR plotting of HM16–HM21 cases. (C) BAF and LRR plotting of HM22–HM27 cases. (D) BAF and LRR plotting of HM28–HM31 cases.
BAF and LRR plotting was visualised by karyoploteR using the data produced with the InfiniumExome-24v1 array (SNP loci number 306,670).

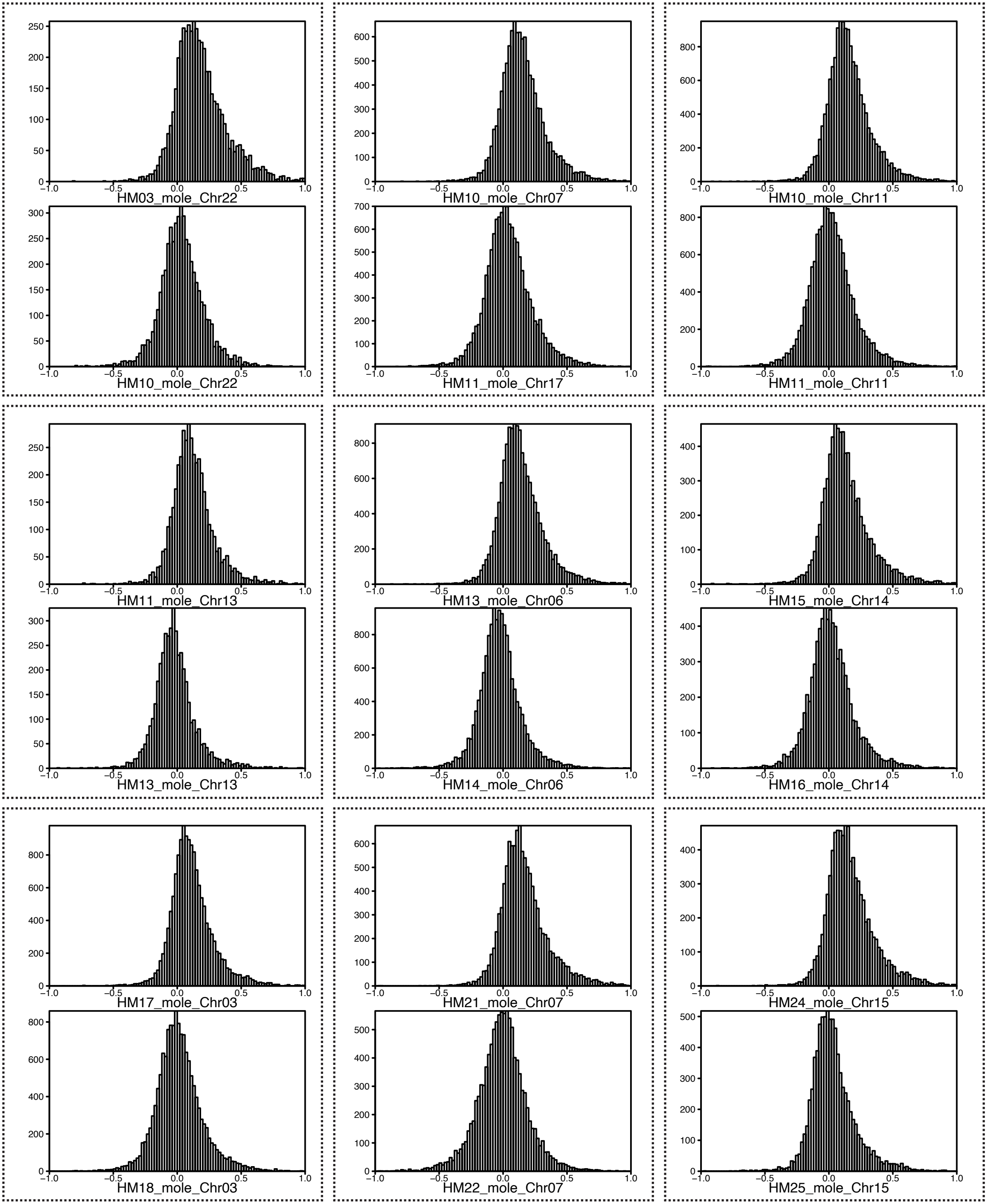

Supplementary Information Figure S2

Supplementary Figure S2

Histograms of log R ratio of trisomic chromosomes.

Upper and lower histograms are from trisomic and disomic chromosomes, respectively. The peaks of histograms of disomic chromosomes are near 1.0. In contrast, the peaks of histograms of trisomic chromosomes shift to the right.

(A)

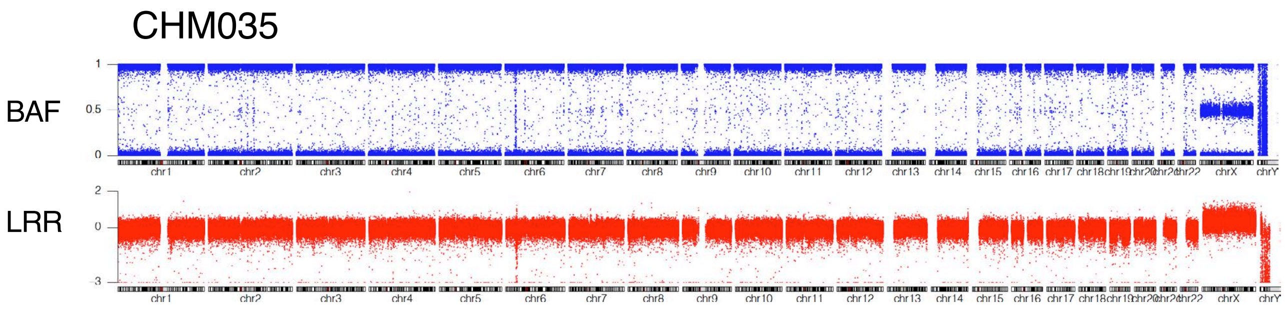

(B)

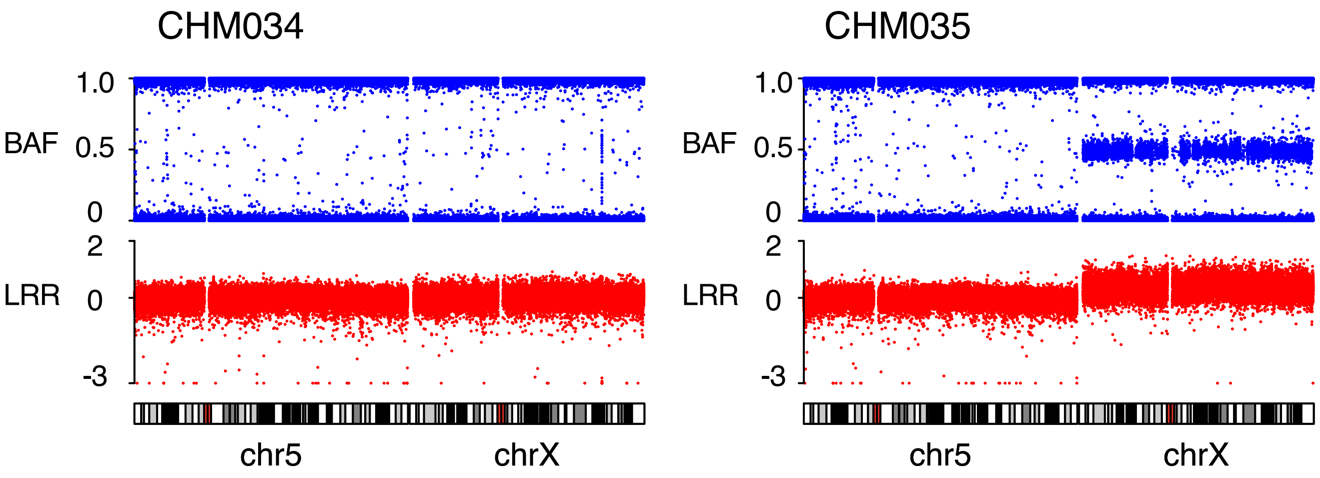

Supplementary Information Figure S3

Supplementary Figure S3

B allele frequency and log R ratio plotting of aneuploid androgenetic monospermic CHM Data are retrieved from GSM1327073 and GSM1327072 on Gene Expression Omnibus (GEO) Datasets in National Center for Biotechnology Information (https://www.ncbi.nlm.nih.gov). (A) B allele frequency (BAF) and log R ratio (LRR) plotting of CHM035. (B) BAF and LRR plotting on chromosomes 5 and X of CHM034 and CHM035. BAF and LRR plotting was visualised by karyoploteR using the data of GPL18247 (Illumina Human1M-Duov3 DNA Analysis BeadChip).
